# Hand hygiene in hospitals: an observational study in hospitals from two southern states of India

**DOI:** 10.1186/s12889-018-6219-6

**Published:** 2018-11-27

**Authors:** Mukta Tyagi, Claudia Hanson, Joanna Schellenberg, Swecha Chamarty, Samiksha Singh

**Affiliations:** 1 Public Health Foundation, Kavuri Hills, Madhapur, Hyderabad 500081 India; 2 Department of Public Health Sciences, Karolinska Institutet, Stockholm, Sweden; 3 Department of Disease Control, London School of Hygiene and Tropical Medicine, London, England

**Keywords:** Hand hygiene, Compliance, Healthcare associated infection

## Abstract

**Background:**

Hand hygiene is a simple and low-cost measure to reduce healthcare associated infection yet it has always been a concern in low as well as high resource settings across the globe. Poor hand hygiene during intra-partum and newborn care may result in sepsis, which is a major cause of death among newborns and puts a financial burden on already strained health systems.

**Methods:**

We conducted non-participatory observations in newborn care units and labour rooms from secondary and tertiary level, public and private hospitals, as part of a baseline evaluation of a quality improvement collaborative across two southern states of India. We assessed hand hygiene compliance during examinations and common procedures, using tools adapted from internationally recommended checklists and World Health Organization’s concept of five moments of hand hygiene. We assessed differences in compliance by type (public/private), level (secondary/tertiary) and case load (low/intermediate/high). Analysis was adjusted for clustering and weighted as appropriate.

**Results:**

We included 49 newborn care units (19 private, 30 public) and 35 labour rooms (5 private, 30 public) that granted permission. We observed 3661 contacts with newborns and their environment, 242 per-vaginal examinations and 235 deliveries. For the newborns, a greater proportion of contacts in private newborn units than public complied with all steps of hand hygiene (44% vs 12%, *p* < 0.001), and similarly in tertiary than secondary units (33% vs 12%, p < 0.001) but there was no evidence of a difference by case load of the facility (low load-28%; intermediate load-14%; high load- 24%, *p* = 0.246). The component with lowest compliance was glove usage where indicated (20%). For deliveries, hand hygiene compliance before delivery was universal in private facilities but seen in only about one-quarter of observations in public facilities (100% vs 27%, *p* = 0.012). Average overall compliance for hand-hygiene during per-vaginal examinations was 35% and we found no evidence of differences by type of facility.

**Conclusion:**

Observed compliance with hand hygiene was low overall, although better in private than public facilities in both newborn units and labour rooms. Glove usage was a particular problem in newborn care units.

**Trial registration:**

Retrospectively registered with Clinical Trials Registry- India (CTRI/2018/04/013014).

**Electronic supplementary material:**

The online version of this article (10.1186/s12889-018-6219-6) contains supplementary material, which is available to authorized users.

## Background

Each year, hundreds of millions of patients are affected by Healthcare-Associated Infections (HAI) worldwide. An estimated 7 of every 100 hospitalized patients in developed and 10 in 100 in low and middle income countries acquire at least one healthcare associated infection causing a major mortality and financial burden on already strained health systems [1]. Healthcare associated infections are those occurring in a patient during the process of care in a hospital or other health-care facility which was not present or incubating at the time of admission. This includes infections acquired in the hospital, but appearing after discharge, and also occupational infections among the staff of the facility [2]. A metanalysis conducted by World Health Organization in the year 2010 found the pooled prevalence of healthcare associated infection to be 15·5 per 100 patients [95% CI 12·6–18·9] in low and middle income countries, which is much higher than that reported in Europe (7.1 per 100 patients) and the USA (4.5 per 100 patients) [3]. The burden of such infections is particularly high in intensive care units for adults [1] and newborns [4].

In India, there are two levels of intensive newborn care units i) Sick Newborn Care Units in secondary public-sector hospitals and ii) Newborn Intensive Care Units in medical colleges and private tertiary care. Septicaemia (a serious bloodstream infection) is the third most common cause of admissions (17%) and mortality (18%) in Sick Newborn Care Units [5]. The rates are higher amongst the newborns referred from other facilities (“out born”) than those born and referred from the labour rooms within the facility (“inborn”) [5]. Septicaemia amongst newborns could be early onset due to transmission of infection from the mother or due to poor hygiene in labour rooms, or late onset due to infection acquired after admission in the newborn care unit.

The contaminated hands of a health care provider could be a source of infections in the admitted newborns [2]. Several studies emphasize the importance of hand hygiene as a simple and effective measure in reduction of such infections [6, 7]. The World Health Organization also issued guidance for a multimodal strategy to improve hand hygiene [2]. A systematic review of 96 studies assessed the prevalence and correlates of compliance with hand hygiene in industrialized countries. It found mean compliance of 40%, lower compliance rates in intensive care units (30–40%) than in other settings (50–60%), among physicians (32%) than nurses (48%), and before (21%) rather than after (47%) patient contact [8]. During our literature search, we found that there is a gap in evidence for association between hand hygiene compliance with the type of facility (public/private); level (secondary/tertiary); and work load of the facility.

This study is part of a baseline evaluation of a quality improvement intervention being implemented in secondary and tertiary care hospitals in two states of India (Andhra Pradesh and Telangana). About 14% of the deaths among inborn and 36% in out born admissions in newborn care units in these states are due to sepsis [5], thus we conducted an assessment to i) measure compliance for hand hygiene practices in newborn care units and labour rooms, and ii) identify the variations in hand hygiene practices by type, level and the load of the facility.

## Methods

### Setting

The study is a cross-sectional assessment and is part of a bigger evaluation of a quality improvement project. [9, 10] All 85 hospitals (public and private) with a newborn care unit (Sick Newborn Care Unit or Newborn Intensive Care Unit) in the states of Telangana and Andhra Pradesh during 2014 were selected for the intervention. Of which 25 had received intervention for more than a year before our assessment. In our study we included the remaining 60 hospitals for the baseline assessment, 25 in Telangana and 35 in Andhra Pradesh. We obtained ethics approval and permissions for the study.

The public healthcare infrastructure in rural India has been developed as a three-tier system based on the population norms [11]. The facilities included in the study were medical colleges, district hospitals, maternity and child health hospitals and area hospitals. These are secondary and tertiary care facilities in the Indian health system. We focused on newborn care units and labour room of these facilities: the health care providers working in these units constitute our study population.

### Data collection

We used cross-sectional data collected by non-participatory observations performed from May 2016 to August 2016. We collected information on number of admissions for the last three months from the date of our visit.

We used three observation checklist tools adopted from internationally recommended checklists [12–15] and utilizing the tool from the World Health Organization concept of five moments of hand hygiene to observe hand-hygiene in newborn care units and labour rooms [12]. The five moment for hand hygiene as mentioned in WHO guidelines are before touching a patient, before clean or aseptic procedures, after risk of exposure to body fluids, after touching a patient and after touching patient surroundings. Separate tools for labour room and newborn care units were developed on an Android based application, linked to the backend server. We used Android based Lenovo tablets for data collection and upload.

The tools were pilot tested in three facilities: two medical colleges and one area hospital, at three different times: twice when we were yet to transfer the tools in mobile application and once after development of the application. The tools and functionality in the application were modified as per findings from these pilots—we fixed the number of hours for observation, simplified data recording, and incorporated drop down options and quality checks. We appointed nursing graduates as observers and trained them extensively for observations and data extraction from registers. Six teams of 4 members each- one supervisor, one field lead and two observers, one for labour room and one for newborn units, were placed in the allocated facilities for six days. In newborn care units, observers spent a minimum of 4 h and in labour rooms a minimum of 6 h every day in direct observation, as per schedule, in either of the two shifts—morning or evening. We kept a longer period of observation in labour rooms so as to be able to observe at least two to three deliveries.

In newborn care units, we observed every contact of a healthcare provider with the admitted newborn for hand hygiene practice during the observation period. The observation unit was thus a contact with the newborn or their environment. However, the number of contacts depended on the severity of sickness as the very sick may get frequent contacts with the healthcare provider. This may also be linked with level of facility where the tertiary hospitals are likely to receive very sick babies. Thus, we adjusted for potential clustering at the facility level due to this sampling method.

In the labour room, we observed 2–3 mothers for hand hygiene compliance during per-vaginal examinations and before conducting delivery, during the 6 h observation period each day. The observers were instructed to observe only one woman at a time even if there was more than one delivery being conducted simultaneously.

Collected data were saved daily and uploaded on a safe server weekly. The data was extracted in MS Excel and checked on a weekly basis. We maximised data quality using several ways: i) inbuilt skips, ranges and checks in the application ii) supervisory visits by lead researchers iii) daily reporting on the number of observations and iv) three levels of data checking exercise- by field supervisors, research assistants and senior investigator.

### Definition of outcomes and explanatory variables

#### Types of contact

We categorized each contact as a patient touch or an environment touch. Environment contact was touching any object or furniture without having touched the patient [16]. This could be the health worker leaning against a bed or a maintenance activity such as changing bed linen. We categorized patient touch as invasive or non-invasive. Invasive contacts are when a contact with blood, body fluids, secretions/excretions, mucous membrane or non-intact skin is made; any other contact was considered as non-invasive [16].

#### Compliance to hand hygiene

Compliance is measured by dividing the number of opportunities where healthcare workers performed hand hygiene following all the necessary steps that ensure interruption of germ transmission by hands (the numerator) to all observed moments when this was required (the denominator). We defined appropriate hand hygiene compliance with respect to type of contact and when all the criteria were met as mentioned in Table 1. In labour room, we only assessed the hand hygiene compliance during per-vaginal examination and delivery. We generated binary variables using the criteria.

**Table 1:** Criteria to be met for defining hand hygiene compliance

Type of contact	Before the contact		After the contact
	Hand-wash with soap and water or waterless alcohol based hand rub	Wearing gloves	Hand-wash with soap and water or waterless alcohol based hand rub
Invasive/Per-vaginal/Delivery	✔	✔	✔
Non-invasive	✔	–	✔
Environment	✔	–	✔

#### Types of facilities

The newborn units were stratified by load of admission based on quartiles (<q1, q1-q3, and > q3). Low load newborn care units have < 35 average admissions per month, medium between 36 and 110 and high load being > 110 admissions per month. Low load labour room facilities have < 67 deliveries per month, medium 67–167 and high load > 167 average deliveries per month [17].

Facilities that provide level 2 newborn care (Special Newborn Care Units) were classified as secondary, and facilities that provide level 3 newborn care (Newborn Intensive Care Units) were considered as tertiary level care for the purpose of analysis [18].

### Data analysis

We used Stata version 14 to generate cross-tabulations [19]. We computed hand hygiene compliance in newborn care units and labour rooms using the definitions mentioned above. The data from newborn care units was considered self-weighted because of universal inclusion of the admitted babies, but clustered with the hospital. We thus adjusted the analysis of this data for clustering while computing proportion of compliance to hand hygiene. For observations in labour room, we weighted the data set for the number of admissions in the labour room and adjusted for clustering within the selected hospital. We computed compliance proportion, confidence interval and also report stratified analysis with the type of facility (private/public), level of facility (secondary/tertiary) and load (low/medium/high). We used chi-square or Fisher’s exact test at 95% confidence level to test any associations. We conducted Poisson regression analysis to test association between compliance to hand hygiene and type of facility adjusting for the level and load of the facility. For this regression analysis, we created a composite variable for level and load of facility combined, i.e. secondary-low, secondary-medium, secondary-high load, likewise tertiary-load.

## Results

### Profile of facilities

**Fig. 1:**
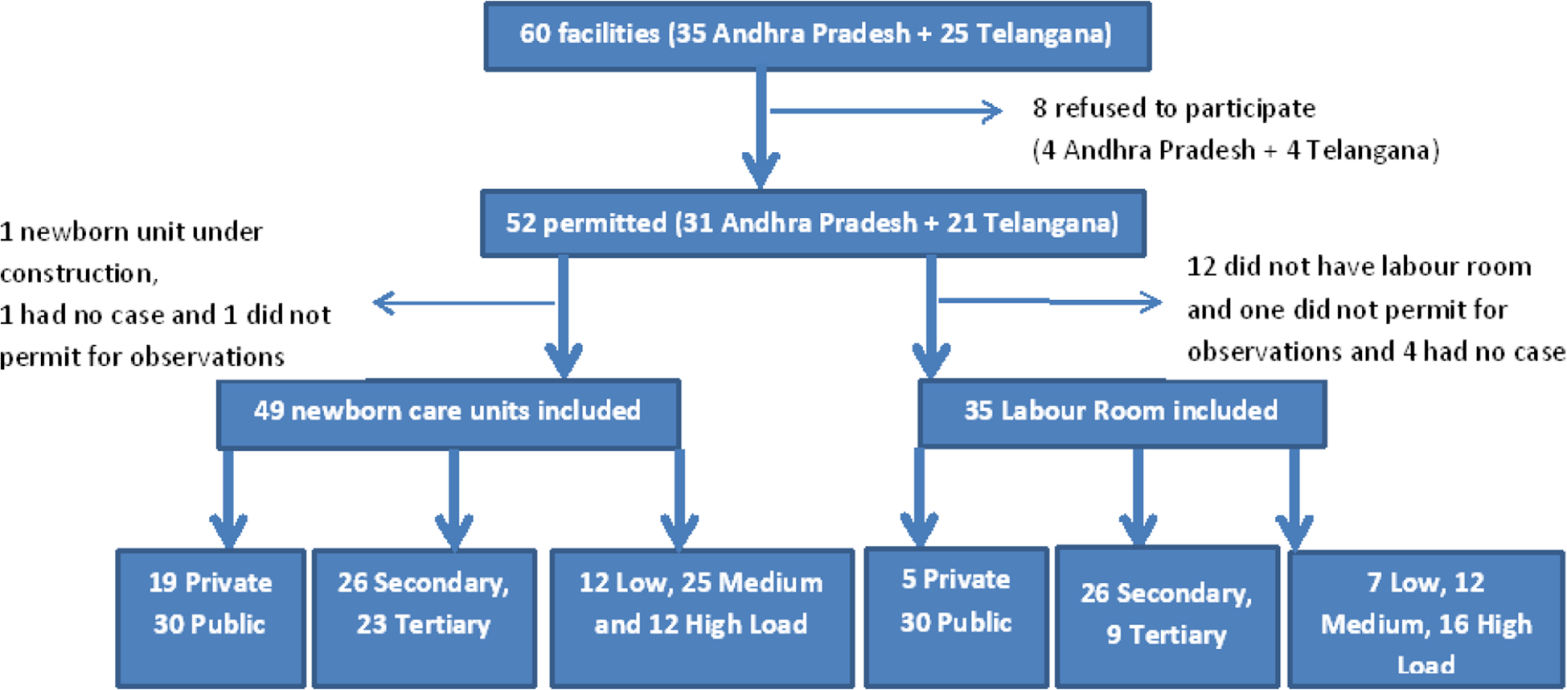
Flow-chart indicating included number of facilities by type, level and case load of the facility

We visited a total of 60 facilities, out of which 52 gave permission for data collection. Out of these 52 facilities, twelve did not have a labour room, one did not give permission for observations and four had no case during our observation period. Therefore, we included 49 newborn care units and 35 labour rooms in our study (Fig. 1). Table 2 gives the number of facilities we visited by type and load. The average number of admissions per month in newborn units (private-median 38, range 4–86; public- median 102, range 4–179) and labour rooms (private-median 58, range 16–159; public- median 157, range 19–979) was almost threefold in public facilities when compared to private. All the private facilities included in the study were tertiary care units for newborn yet none of these had a high case load.

**Table 2:** Distribution of facilities by type, level and admission load for newborn care and obstetric care

	Newborn Care		Obstetric Care	
No. of facilities visited	Private *N* = 19	Public *N* = 30	Private *N* = 5	Public *N* = 30
Number of facilities by Level of facility^a^				
Secondary	0	26	0	26
Tertiary	19	4	5	4
Average admission per month				
Mean (SE)	39 (5.32)	102 (9.03)	73 (34.88)	235 (40.95)
Median	38	102	58	157
Range	4–86	4–179	16–159	17–979
Number of facilities by Case load				
Low	9	3	2	5
Medium	10	15	2	10
High	0	12	1	15

^a^Secondary-Area Hospital, Maternity and Child Hospital, District Hospital and General Hospital, Tertiary- Government, Medical College, Private Medical College, Private Specialty/ Multi-specialty

There were no secondary level private facilities, and no high load newborn units in private facilities

### Availability of hand-hygiene protocols

A hand-hygiene protocol was available in 98% of the newborn care units and 86% reported that the protocols were adopted from national or international standards (Table 3). While 72% public facilities displayed protocols, 94% private facilities did so. A lesser percentage of public (72%), and secondary facilities (67%) displayed the protocol in comparison to private (94%) and tertiary level facilities (95%), respectively.

**Table 3:** Percentage facilities with availability of hand hygiene protocols in newborn care units and Labour room^a^

	Total	Type		Level		Load		
		Private	Public	Secondary	Tertiary	Low	Medium	High
	*N* = 44, %	*N* = 17, %	*N* = 27, %	*N* = 23, %	*N* = 21, %	*N* = 9, %	N = 23, %	*N* = 12, %
Available in newborn units	98	100	96	96	100	100	96	92
Adopted from standards	86	81	88	91	80	89	77	91
Written guidelines	88	87	88	91	85	78	86	91
Protocols displayed	81	94	72	67	95	89	77	73
	*N* = 35, %	*N* = 5, %	*N* = 30, %	*N* = 26, %	*N* = 9, %	*N* = 7, %	*N*-12, %	*N* = 16, %
Available in labour rooms	88	80	90	88	89	71	100	87
Adopted from standards	68	50	71	74	50	20	83	71
Written guidelines	64	75	63	61	75	80	75	50
Protocols displayed	71	100	67	65	87	80	75	64

*N is the total number of facilities in each group*

^*a*^ *Information not available for five facilities out of forty nine*

Hand-hygiene protocol for labour room was available in 88% of the facilities, but only 68% of the available protocols were adapted from national or international standards and 71% of potentially available protocols were displayed at a prominent place.

### Hand-hygiene compliance in newborn care units

We observed a total of 3661 contacts with the patient and/or their environment (Table 4). Out of the total, 3032 were direct contacts with the patients. Percentage compliance by type, level and load of facility and, component of hand-hygiene is presented in Table 4.

**Table 4:** Percentage compliance for hand-hygiene in newborn care units by type, level and load of facility

	TOTAL COMPLIANCE % [95%CI]	Type			Level			Load^1^			
		Private % [95%CI]	Public % [95% CI]	*P* value	Secondary % [95% CI]	Tertiary % [95% CI]	*P* value	Low load % [95% CI]	Medium load % [95% CI]	high load % [95% CI]	*P* value
	*N* = 3661	*N* = 1201	*N* = 2460		*N* = 1838	*N* = 1823		*N* = 2069	*N* = 1108	*N* = 309	
TOTAL COMPLIANCE %	23 [15–32]	44 [33–56]	12 [6–26]	< 0.001	12 [4–30]	33 [23–46]	0.037	28 [18–41]	14 [5–31]	24 [8–53]	0.246
Without any invasive procedure^2^	*n* = 1556	*n* = 470	*n* = 1086		*n* = 801	*n* = 755		*n* = 660	*n* = 698	*n* = 170	
Overall compliance	21 [12–32]	45 [26–65]	10 [4–21]	0.001	7 [3–18]	35 [19–54]	0.002	25 [13–42]	13 [4–31]	28 [8–62]	0.367
Hand wash prior to contact	44 [31–59]	66 [37–87]	35.0 [21–51]	0.059	24 [13–38]	66 [48–81]	0.001	46 [26–68]	36 [19–59]	64 [56–72]	0.279
Hand wash after contact	39 [28–51]	58 [46–70]	30 [18–45]	0.005	24 [12–42]	54 [40–67]	0.009	46 [35–56]	25 [12–46]	58 [33–80]	0.361
Contact with blood, body fluids and invasive procedures	*n* = 1476	*n* = 454	*n* = 1022		*n* = 730	*n* = 746		*n* = 945	*n* = 300	*n* = 105	
Overall compliance	14 [8–24]	37 [24–53]	4 [2–9]	< 0.001	2 [1–5]	26 [15–41]	< 0.001	14 [7–27]	16 [4–45]	23 [10–44]	0.739
Hand wash prior to contact	37 [25–52]	65 [44–82]	25 [14–41]	0.002	22 [9–45]	52 [35–70]	0.036	42 [26–59]	28 [10–55]	49 [32–66]	0.393
Hand wash after contact	39 [29–50]	65 [50–78]	27 [18–39]	< 0.001	21 [11–35]	57 [42–70]	< 0.001	40 [29–53]	31 [14–55]	64 [33–86]	0.265
Glove usage	20 [13–31]	45 [29–62]	10 [5–17]	< 0.001	6 [3–10]	35 [22–51]	< 0.001	19 [10–32]	25 [9–52]	45 [38–33]	0.192
Environment contact	*n* = 629	*n* = 277	*n* = 352		*n* = 307	*n* = 322		*n* = 464	n = 110	*n* = 34	
Overall compliance	48 [27–69]	53 [31–75]	44 [14–78]	0.669	48 [16–82]	48 [26–70]	0.009	61 [38–79]	13 [5–26]	9 [2–28]	< 0.001
Hand wash prior to contact	62 [42–79]	75 [51–89]	53 [21–82]	0.278	57 [23–86]	67 [44–85]	0.642	76 [56–89]	20 [11–34]	26 [15–42]	< 0.001
Hand wash after contact	58 [38–75]	60 [37–79]	56 [26–82]	0.853	57 [24–85]	59 [38–77]	0.852	68 [46–84]	30 [13–55]	26 [13–47]	0.011

*N is the total number of observations in each group*

^*1*^ *One hospital did not permit for collection of data on the number of admissions for the last three months*

^*2*^ *Assuming that a) Observers were aware of the fact that gloves are required when a contact with blood, body fluids or for some invasive procedure is made. b) They appropriately ticked the options*

In only 23% of the contacts, hand-hygiene compliance was followed as per the standards with marked differences between public (12%) and private facilities (44%), *p* < 0.001. Similarly, there was a significant difference in compliance by secondary and tertiary facilities (12 and 33%; *p* = 0.037). We found no evidence of a difference, however, for low, medium and high load facilities (28, 14, and 24%; *p* = 0.246).

**Fig. 2:**
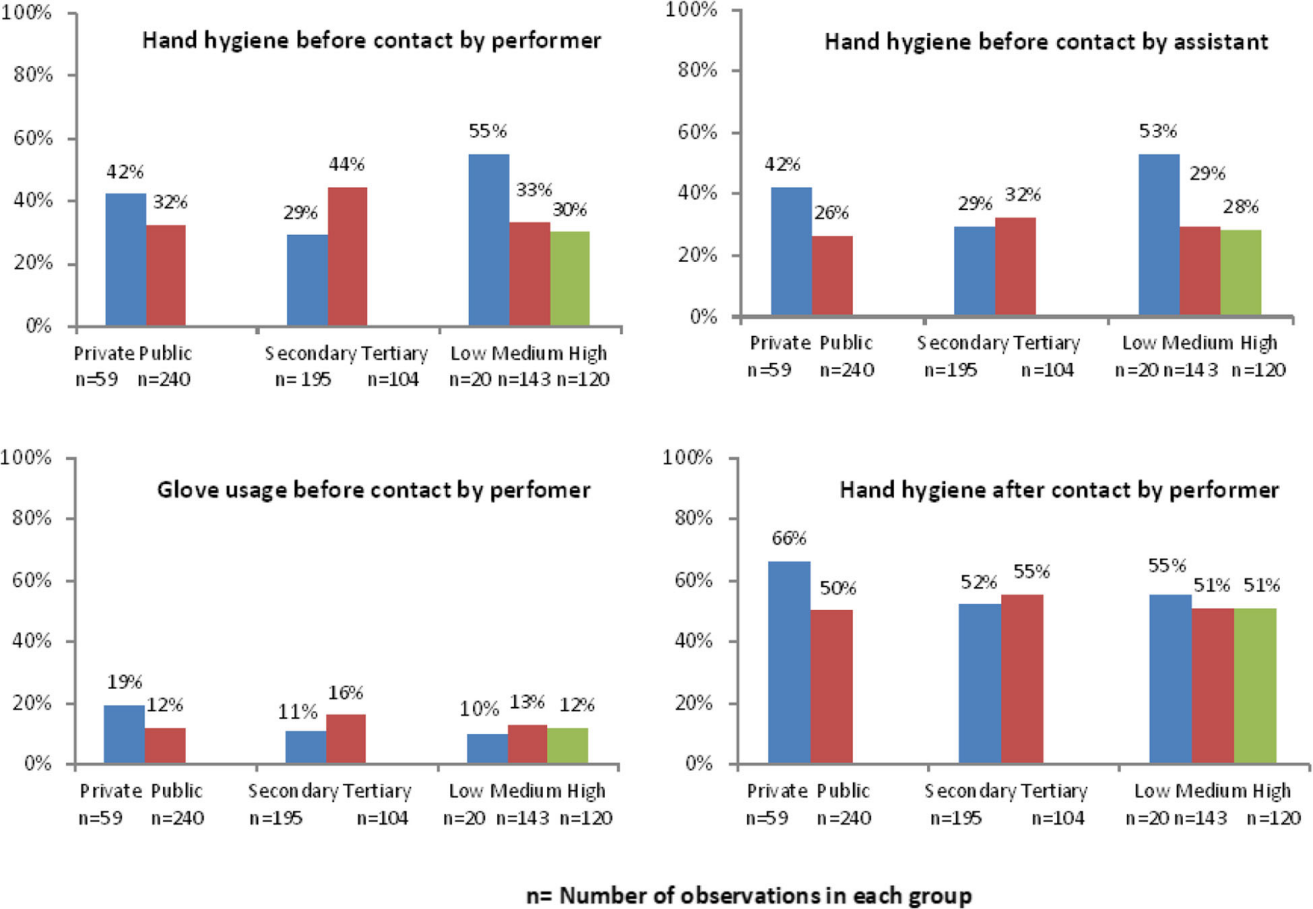
Percentage compliance for hand-hygiene during invasive procedures on newborn by type, level and load of facility

Hand-hygiene compliance was better for environment contact (48%) than for patient contacts (non-invasive-21%, invasive or contact with blood or body fluids-14%). Among contacts for invasive procedures, we found better overall compliance in private than public facilities (17, 3%; *p* = 0.004) and in tertiary than secondary facilities (10, 2%; *p* = 0.048) (Additional file 1: Table S1). We found no evidence of a difference in overall compliance during invasive procedures by load of the facility. Poor glove usage (20%) during invasive contacts was the biggest contributory factor to overall low compliance. The poorest compliance was observed in glove usage by the person performing the procedure (13%) whereas hand hygiene after a procedure by the performer was most commonly complied with (51%). Figure 2 depicts the compliance to various components of hand hygiene during invasive procedures, with type, level and load of facility.

### Hand-hygiene compliance in labour rooms

We observed 242 per-vaginal examinations during intrapartum care and 235 deliveries. Overall compliance for hand-hygiene for per-vaginal examinations was 35% (Additional file 2: Table S2) and 34% for delivery (Table 5). In all the observed per-vaginal examinations and deliveries, glove usage was adhered to in all the cases. The attributing factor for a low percentage of compliance was hand wash before the procedures (38% during per-vaginal examinations during intrapartum care; 34% before delivery).

**Table 5:** Percentage compliance for hand-hygiene during delivery by type, level and load of facility, % [95% Confidence Interval]

	TOTAL COMPLIANCE	Type			Level			Load			
		Private, % [95%CI]	Public, % [95%CI]	*P* value	Secondary, % [95%CI]	Tertiary, % [95%CI]	*P* value	Low load, % [95%CI]	Medium load, % [95%CI]	high load, % [95%CI]	*P* value
	*N* = 235	*N* = 8	*N* = 227		*N* = 194	*N* = 41		*N* = 11	*N* = 51	*N* = 173	
TOTAL compliance	34 [20–51]	100	27 [14–45]	0.012	29 [15–49]	51 [20–81]	0.259	47 [14–82]	43 [19–71]	23 [8–49]	0.403
Hand-wash before delivery	34 [20–51]	100	27 [14–45]	0.011	29 [15–49]	51 [20–81]	0.259	47 [14–82]	43 [19–71]	23 [8–49]	0.403
Wore gloves	100	100	100		100	100		100	100	100	

*N is the total number of observations in each group*

On adjusting for level and the load of the facility, we found that newborn care units in private facilities have 7.26 (5.47–9.63) times higher hand hygiene compliance than public (*p* < 0.001). In labour room, private facilities have 7.09 (2.39–20.97) times higher compliance than public (*p* = 0.001). However, only five private facilities had labour rooms thus power of this result is likely to be less.

## Discussion

We found overall hand hygiene compliance to be 23% in newborn care units, 44% in private and 12% in public facilities. Compliance was less in secondary facilities (12%) compared to tertiary facilities (33%). In labour wards, compliance for hand hygiene before conducting delivery was better in private (100%) than public (27%) while levels were similar in secondary and tertiary facilities.

Our finding that compliance to hand hygiene is influenced by facility ownership: better in private than public, has also been reported by a recent study conducted in China. It reported compliance in 77 private and 152 public hospitals with compliance to be significantly better in private than public (79, 67%, *p* < 0.05) [20].

We found a compliance of 33% in tertiary newborn care facilities which is slightly lower than reported from other parts of India that reported hand hygiene compliance levels of 43% of 911 hand hygiene opportunities in Punjab in 2011 [21] and 46% of 15,797 observed opportunities in Delhi in 2015 [22]. Mortality among inborn special newborn care units admissions in year 2013–15 is 4% in Punjab, 6% in Delhi and 8% in Andhra Pradesh [5]. We found compliance for hand wash before conducting delivery to be 34% (2016) whereas a 2012 pilot study in one sub-district hospital in Karnataka observed it to be as low as 11% on 388 observations in the delivery ward during intrapartum care [23]. The states included in the current study are, like Karnataka, part of southern India and are very similar in health indicators. In the year 2014, the health ministry launched the Indian New Born Action Plan [24] that laid increased focus on hand-washing with soap and water during birth practices. This could have influenced the better hand hygiene compliance in our study from 2016 compared to that from Karnataka in 2012. In a 2016 study from Rajasthan where primary health care facilities were also included, the hand hygiene compliance during childbirth was 2% in 240 observed cases at baseline [25] which is very low in comparison to our study (34%) that included only secondary and tertiary level facilities.

Poor hand hygiene adherence has long been a concern not only in low [17, 20, 21, 25] but also in high resource settings across the globe [8, 26] . Hand hygiene compliance is reported as ranging from 8 to 39% in Sub-Saharan African countries [27–29]. The studies from Asian countries report compliance to be ranging between 18 and 46% [20, 30, 31]. A systematic review of ninety-six studies from industrialized countries reported median compliance rates for intensive care units to be in the range of 30–40% [8].

Many studies and reports have identified factors affecting compliance. Knowledge of hand hygiene practice, training, availability of essential logistics for maintaining hand hygiene and knowledge of presence of infection prevention committees are the factors that influence hand hygiene compliance [20, 32–34] . High workload was mentioned as one of the reasons for non-compliance by 38% of 100 health workers in a study conducted in Pune [35], while another study conducted in a Delhi pediatric intensive care unit observed 100 hand hygiene sessions and reported a decrease in compliance with increased workload [36]. In our study, we did not find any significant variation by load of the facility.

To the best of our knowledge, this study is the first to assess the hand hygiene compliance focused on different characteristics of the facilities in a large number of Indian hospitals. We used direct observation for assessment of hand-hygiene compliance as recommended by WHO [16]. We observed hand hygiene during various shifts spread over six days so that the Hawthorne effect [37] is likely to be minimized. We did extensive training of observers to maximise data quality. There were a few limitations to our study. We missed the opportunity to record hand hygiene after delivery as the health worker got involved in multiple activities post childbirth and we failed to track that. We did not collect data on hand-hygiene-related knowledge and attitude of health care workers and on the availability of the logistics and infrastructure required for hand hygiene. We also did not record the cadre of healthcare worker (physician/nurse/ward staff) making the contact.

Several interventions have been tested to improve hand-hygiene compliance in different settings [22, 38–40]. A quality improvement initiative has already been initiated in a set of our study hospitals. We plan to conduct another assessment towards the end of intervention to study the effect.

## Conclusions

This study provides evidence that hand hygiene compliance was low in newborn care units and during intrapartum care in labour rooms from the two states. Compliance is poorer in public hospitals in both the newborn care units and labour rooms compared to private hospitals. In newborn care units, glove usage was the least followed step. Improving the availability and display of written hand hygiene protocols; supervision and feedback; and quality improvement initiatives could be useful methods to improve compliance.

## Additional files

Additional file 1: **Table S1.** Percentage compliance for hand-hygiene during invasive procedures on newborn by type, level and load of facility. N is the total number of observations in each group. ^*1*^*One hospital did not permit for collection of data on the number of admissions for the last three months.* ^*2*^*Person performing the procedure.* ^*3*^*Person assisting performer in the procedure.* (DOCX 50 kb)

Additional file 2: **Table S2.** Percentage compliance for hand-hygiene during per-vaginal examinations by type, level and load of facility. *N is the total number of observations in each group.* ^*1*^*One hospital did not permit for collection of data on the number of admissions for the last three months* (DOCX 50 kb)

## Abbreviations

WHO: World Health Organization
