# Supplementary material for: Hand hygiene in hospitals: an observational study in hospitals from two southern states of India
Source: BMC Public Health. 2018 Nov 27;18:1299. doi: 10.1186/s12889-018-6219-6 (PMC6257976; doi:10.1186/s12889-018-6219-6)
Supplement: Supplementary file 1 — Table S1. Percentage compliance for hand-hygiene during invasive procedures on newborn by type, level and load of facility. N is the total number of observations in each group. 1One hospital did not permit for collection of data on the number of admissions for the last three months. 2Person performing the procedure. 3Person assisting performer in the procedure. (DOCX 50 kb) [file 12889_2018_6219_MOESM1_ESM.docx]

**Supplementary Table 1: Percentage compliance for hand-hygiene during invasive procedures on newborn by type, level and load of facility**

|  | **TOTAL COMPLIANCE**  **%**  **[95%CI]** | **Type** | | | **Level** | | | **Load** | | | |
| --- | --- | --- | --- | --- | --- | --- | --- | --- | --- | --- | --- |
|  |  | **Private**  **%**  **[95%CI]** | **Public**  **%**  **[95%CI]** | **P value** | **Secondary**  **%**  **[95%CI]** | **Tertiary**  **%**  **[95%CI]** | **P value** | **Low load %**  **[95%CI]** | **Medium load^[[1]](#footnote-1)^**  **%**  **[95%CI]** | **high load**  **%**  **[95%CI]** | **P value** |
|  | **N=299** | **N=59** | **N=240** |  | **N=195** | **N=104** |  | **N=20** | **N=143** | **N=120** |  |
| **TOTAL COMPLIANCE** | **5**  **[2-11]** | **17**  **[4-49]** | **3**  **[1-6]** | **0.004** | **2**  **[1-7]** | **10**  **[3-28]** | **0.048** | **5**  **[1-34]** | **7**  **[2-22]** | **2**  **[1-9]** | **0.432** |
| Hand hygiene before contact by performer^[[2]](#footnote-2)^ | 34  [24-46] | 42  [11-81] | 32  [23-43] | 0.462 | 29  [18-42] | 44  [22-69] | 0.238 | 55  [21-85] | 33  [16-55] | 30  [20-43] | 0.767 |
| Hand hygiene before contact by assistant^[[3]](#footnote-3)^ | 30  [19-44] | 42  [10-82] | 26  [16-41] | 0.462 | 29  [16-46] | 32  [14-57] | 0.813 | 53  [17-86] | 29  [15-49] | 28  [10-56] | 0.589 |
| Glove usage by performer^2^ | 13  [8-21] | 19  [5-51] | 12  [7-20] | 0.135 | 11  [6-21] | 16  [7-35] | 0.264 | 10  [2-34] | 13  [6-25] | 12  [4-27] | 0.879 |
|  |  |  |  |  |  |  |  |  |  |  |  |
| Hand hygiene after contact by performer^2^ | 51  [35-67] | 66  [47-81] | 50  [30-69] | 0.228 | 52  [30-73] | 55  [38-71] | 0.834 | 55  [19-86] | 51  [31-71] | 51  [23-78] | 0.980 |
|  |  |  |  |  |  |  |  |  |  |  |  |

1. [↑](#footnote-ref-1)
2. [↑](#footnote-ref-2)
3. [↑](#footnote-ref-3)
