# Supplementary material for: Hand hygiene in hospitals: an observational study in hospitals from two southern states of India
Source: BMC Public Health. 2018 Nov 27;18:1299. doi: 10.1186/s12889-018-6219-6 (PMC6257976; doi:10.1186/s12889-018-6219-6)
Supplement: Supplementary file 2 — Table S2. Percentage compliance for hand-hygiene during per-vaginal examinations by type, level and load of facility. N is the total number of observations in each group. 1One hospital did not permit for collection of data on the number of admissions for the last three months (DOCX 50 kb) [file 12889_2018_6219_MOESM2_ESM.docx]

**Supplementary Table 2: Percentage compliance for hand-hygiene during per-vaginal examinations by type, level and load of facility**

|  | **TOTAL COMPLIANCE**  **%**  **[95%CI]** | **Type** | | | **Level** | | | **Load^1^** | | | |
| --- | --- | --- | --- | --- | --- | --- | --- | --- | --- | --- | --- |
|  |  | **Private**  **%**  **[95%CI]** | **Public**  **%**  **[95%CI]** | **P value** | **Secondary**  **%**  **[95%CI]** | **Tertiary**  **%**  **[95%CI]** | **P value** | **Low load**  **%**  **[95%CI]** | **Medium load**  **%**  **[95%CI]** | **high load**  **%**  **[95%CI]** | **P value** |
|  | **N=242** | **N=21** | **N=221** |  | **N=175** | **N=67** |  | **N=14** | **N=73** | **N=155** |  |
| **TOTAL COMPLIANCE** | **35**  **[20-54]** | **67**  **[25-92]** | **32**  **[14-51]** | **0.097** | **33**  **[16-57]** | **40**  **[15-70]** | **0.725** | **33**  **[7-75]** | **52**  **[23-80]** | **24**  **[8-52]** | **0.352** |
| Hand wash before per- vaginal examination | 38  [22-56] | 67  [25-92] | 32  [16-53] | 0.128 | 37  [19-60] | 40  [15-70] | 0.874 | 33  [7-75] | 60  [31-83] | 24  [8-53] | 0.190 |
| Glove usage before per- vaginal examination | 100 | 100 | 100 |  | 100 | 100 |  | 100 | 100 | 100 |  |
| Hand wash after per vaginal examination | 60  [43-75] | 80  [28-98] | 56  [43-75] | 0.333 | 59  [39-77] | 61  [32-85] | 0.909 | 50  [15-85] | 75  [42-92] | 54  54 | 0.479 |
